# Association of an inflammaging score based on IL-6, IL-10 and CXCL9, and frailty with long-term mortality in hospitalized older adults

**DOI:** 10.1186/s12979-025-00553-5

**Published:** 2025-12-19

**Authors:** Matilde Sbriscia, Sonia Fantone, Mirko Di Rosa, Francesca Marchegiani, Rina Recchioni, Giulia Matacchione, Chiara Giordani, Francesco Piacenza, Robertina Giacconi, Davide Gentilini, Luciano Calzari, Carlo Fortunato, Gretta Veronica Badillo Pazmay, Monia Cecati, Sara Caccese, Emanuele Francini, Rosanna Maniscalco, Maurizio Cardelli, Elena Tortato, Federica Lenci, Yuri Rosati, Maurizio Burattini, Roberto Antonicelli, Andrea Corsonello, Luca Soraci, Leonardo Biscetti, Massimiliano Fedecostante, Riccardo Sarzani, Marco Malavolta, Tiziana Casoli, Maria Conte, Silvia Di Valerio, Angelica Giuliani, Antonio Domenico Procopio, Fabrizia Lattanzio, Anna Rita Bonfigli, Antonio Cherubini, Claudio Franceschi, Jacopo Sabbatinelli, Fabiola Olivieri

**Affiliations:** 1 Advanced Technology Center for Aging Research and Geriatric Mouse Clinic, IRCCS INRCA, Ancona, Italy; 2 Centre for Biostatistics and Applied Geriatric Clinical Epidemiology, IRCCS INRCA, Ancona, Italy; 3 Clinic of Laboratory and Precision Medicine, IRCCS INRCA, Ancona, Italy; 4 Bioinformatics and Statistical Genomics Unit, Istituto Auxologico Italiano IRCCS, Cusano Milanino, Italy; 5 Department of Brain and Behavioral Sciences, University of Pavia, Pavia, Italy; 6 Scientific Direction, IRCCS INRCA, Ancona, Italy; 7 Diabetology Unit, IRCCS INRCA, Ancona, Italy; 8 Unit of Nephrology and Dialysis, IRCCS INRCA, Ancona, Italy; 9 Respiratory Medicine, IRCCS INRCA, Osimo, Italy; 10 Internal Medicine, IRCCS INRCA, Osimo, Italy; 11 Cardiology Unit, IRCCS INRCA, Ancona, Italy; 12 Unit of Geriatric Medicine, IRCCS INRCA, Cosenza, Italy; 13 Department of Pharmacy, Health and Nutritional Sciences, University of Calabria, Rende, Italy; 14 Neurology Unit, IRCCS INRCA, Ancona, Italy; 15 Geriatria, Accettazione Geriatrica e Centro Di Ricerca Per L’invecchiamento, IRCCS INRCA, Via Tronto 10/A, 60126 Ancona, Italy; 16 Department of Clinical and Molecular Sciences (DISCLIMO), Università Politecnica Delle Marche, Ancona, Italy; 17 Internal Medicine and Geriatrics, Hypertension Excellence Centre’ of the European Society of Hypertension, Società Italiana Per Lo Studio Dell’Aterosclerosi (SISA) LIPIGEN Centre, IRCCS INRCA, Ancona, Italy; 18 Center for Neurobiology of Aging, IRCCS INRCA, Ancona, Italy; 19 Department of Medical and Surgical Sciences (DIMEC), University of Bologna, Bologna, Italy; 20 Institute of Biogerontology, Lobachevsky State University, Nizhny Novgorod, Russia

**Keywords:** CXCL9, Interleukin-6, Interleukin-10, Frailty, Mortality

## Abstract

**Background:**

Aging is accompanied by chronic low-grade inflammation ("inflammaging"), which contributes to increased morbidity and mortality in older adults. This study evaluated the prognostic value of circulating inflammatory biomarkers, i.e. interleukin-6 (IL-6), interleukin-10 (IL-10), and CXCL9, and their integration with frailty for long-term risk stratification.

**Methods:**

We analyzed 1,009 patients (median age, 84 years) hospitalized in acute care wards of three Italian geriatric hospitals as part of the ReportAGE cohort. Frailty was assessed using a deficit accumulation–based Frailty Index (FI), and serum cytokines were measured by immunoassay. Cytokine-specific risk categories were combined into a composite I3 score (range, 3–9). Cox proportional hazards models adjusted for age, sex, comorbidity burden, polypharmacy, and laboratory variables were used to assess associations with 10-year mortality. In a subset of 237 patients, DNA methylation–based estimates of cytokine levels were also analyzed.

**Results:**

Higher I3 scores were independently associated with increased mortality (hazard ratio [HR] 2.42, 95% CI 1.74–3.37 for high vs. low scores), with CXCL9 as the strongest individual predictor (HR 1.69, 95% CI 1.30–2.19). The I3 score improved risk prediction beyond FI alone, and their combination identified four distinct risk groups, with the highest mortality observed among patients with both elevated FI and I3 scores. The integrated model (Clinical variables + FI + I3) achieved the greatest discrimination during the first seven years of follow-up. Serum IL-6 and CXCL9 correlated with their DNA methylation–based estimates, supporting an epigenetic contribution to chronic inflammation.

**Conclusions:**

The I3 score complements frailty assessment and enhances long-term mortality prediction in hospitalized older adults.

**Supplementary Information:**

The online version contains supplementary material available at 10.1186/s12979-025-00553-5.

## Introduction

Inflammaging and frailty are two hallmark manifestations of the aging process, representing the biological and clinical complexity of age-related decline [1]. Inflammaging refers to a chronic, low-grade, systemic proinflammatory state that emerges with age [2–4]. Frailty, on the other hand, is a clinical syndrome of increased vulnerability and diminished resilience to stressors, leading to a higher risk of adverse outcomes [5]. Both conditions are strongly associated with unhealthy aging and increased mortality.

Identifying reliable, minimally invasive biomarkers of inflammaging, and understanding their relationship with frailty, is critical to improving patient stratification in geriatric care. However, real-world data in older adults with multimorbidity and polypharmacy remain limited [6].

Among the most studied inflammatory markers is interleukin-6 (IL-6), a pleiotropic cytokine involved in immune regulation and inflammation. IL-6 levels increase with age and are linked to frailty, functional decline, and mortality [6–9]. Interleukin-10 (IL-10), part of the senescence-associated secretory phenotype (SASP), has also been implicated in immunosenescence and poor outcomes in older adults [10, 11]. It acts mainly as an anti-inflammatory cytokine but can also display context-dependent pro-inflammatory effects, which may result in immune paralysis in chronic conditions [12]. CXCL9, also known as monokine induced by interferon-γ (MIG), has recently emerged as a novel biomarker of inflammaging. Its age-associated expression is linked to endothelial dysfunction, cellular senescence, and cardiovascular aging [10, 11]. Elevated circulating CXCL9 has been observed in patients with cardiovascular disease, in ostensibly healthy older adults, and in association with frailty and mortality risk [13–15]. Crucially, CXCL9 emerged as the strongest contributor to iAge, an inflammatory clock of aging that displayed associations with multimorbidity, frailty, cardiovascular aging, and exceptional longevity in centenarians [11].

Advances in immunosenescence research have led to the development of immunological ageing clocks These predictive models estimate biological age based on immune system parameters, such as cytokine profiles and functional markers, providing insights into immunosenescence and age-related disease risk. The main aim is to quantify inflammaging and predict clinical outcomes independently of chronological age [11, 16]. These approaches reaffirm the central role of IL-6 and identify CXCL9 as a key regulator of inflammatory and senescence-associated pathways [16, 17]. Elevated IL-6 and CXCL9 levels have been linked to accelerated immunological ageing and adverse outcomes in patients with chronic age-related diseases, including end-stage renal disease [18].

DNA methylation has also emerged as a central hallmark of ageing. Specific and reproducible changes in CpG methylation patterns accumulate over time, reflecting both environmental exposures and intrinsic biological processes [19].

Although frailty has been widely adopted to stratify mortality risk following hospitalization [20], few studies have examined its integration with inflammaging biomarkers in this context. In the present study, we assessed frailty using a Frailty Index (FI) based on the deficit accumulation model proposed by Rockwood and colleagues [21], alongside selected inflammaging biomarkers – IL-6, IL-10, and CXCL9. We also developed a novel composite biomarker score, the “I3 score,” and evaluated their associations with 10-year mortality in a real-world cohort of hospitalized older adults. In a subgroup of patients, we analyzed DNA methylation of IL-6, IL-10, and CXCL9 to explore their epigenetic regulation. Our aim was to evaluate the prognostic significance of these biomarkers and improve risk stratification, providing new insights into the clinical relevance of inflammaging in geriatric medicine.

## Methods

### Study design

This was a prospective observational study based on data from the Report-AGE project (ClinicalTrials.gov identifier: NCT01397682), an ongoing epidemiological cohort focused on hospitalized older adults [22]. Patients aged ≥ 65 years admitted to acute care wards (geriatric medicine, cardiology, urology, surgery, neurology) of three Italian IRCCS INRCA hospitals between September 2011 and October 2021 were consecutively enrolled. During the enrolment period, more than 5,000 patients were recruited, and over 90% of eligible individuals provided informed consent to participate. The present analysis was a predefined substudy including approximately 1,000 patients for whom biological samples were collected and stored under standardized protocols.

The study was conducted in accordance with the Declaration of Helsinki and approved by the Ethics Committees of the IRCCS INRCA network. All participants provided written informed consent prior to enrollment. Only patients able to provide informed consent were included, which may have led to underrepresentation of individuals with severe cognitive impairment or impaired consciousness.

### Participants

Inclusion criteria were: age ≥ 65 years, hospital stay ≥ 24 h, and informed consent. Patients with hematological malignancies or active COVID-19 were excluded. A comprehensive geriatric assessment (CGA) was conducted at admission and discharge using the InterRAI Minimum Data Set for Acute Care (MDS-AC). Patients who died during hospitalization were excluded from this analysis to minimize bias from acute conditions. Mortality status was obtained from hospital discharge records and regional registries. Therefore, active follow-up of individual patients was not required and no cases were lost to follow-up.

Comorbidities were recorded and coded according to the International Classification of Diseases, Ninth Revision (ICD-9), as follows: AMI: 410–414, CHF: 428; CeVD: 430–438; Dementia: 290; 331; 294; Depression: 296; COPD: 491; 492; 494; 496; Parkinson: 332; Hypertension: 401–405; CKD: 585; 586; 587; Anemia: 280–285; 790; 6842; Diabetes: 2500–2507; Cancer: 140–172; 174–195; 200–208. The category ‘Dementia’ referred to patients with mild to moderate cognitive impairment, with preserved decisional capacity and ability to provide informed consent. Multimorbidity was assessed using the Charlson Comorbidity Index (CCI). Frailty was quantified using a Frailty Index (FI) based on the deficit accumulation model [21]. Frailty criteria were abstracted from the electronic medical record and standardized clinical assessments documented during hospitalization; self-reported items were not used. Thirty health deficits were included (e.g., comorbidities, functional impairments, cognitive and sensory deficits, polypharmacy), and the Frailty Index (FI) was calculated as the ratio of deficits present to the total number of items (30), yielding scores from 0 (no deficits) to 1 (maximum deficits) (Supplemental Table 1). FI was categorized as non-frail (< 0.10), prefrail (0.10–0.19), and frail (≥ 0.20) based on previously proposed thresholds [5]. Performance-based measures (e.g., grip strength) were not analyzed owing to missingness at admission and the primary use of the deficit-accumulation FI framework.

### Biomarker assessment

Serum levels of IL-6, IL-10, and CXCL9 were measured in triplicate using ELLA™ microfluidic immunoassays (ProteinSimple, Bio-Techne, USA) from fasting samples collected within 48 h of admission. Cytokine concentrations were determined using a customized 32-plex cartridge. Limits of detection (LoD) were: IL-6 (0.11 pg/mL), IL-10 (0.17 pg/mL), and CXCL9 (8.8 pg/mL). Intra- and inter-assay coefficients of variation were all below 11%. For biomarker concentrations below the LOD, values were imputed as LOD divided by the square root of 2 (LOD/√2).

### Epigenetic assessment of cytokines: model-based estimates and locus-specific methylation

In a subgroup of 237 participants, DNA methylation profiles from peripheral white blood cells were analyzed using the Infinium HumanMethylationEPIC v1.0 BeadChip (Illumina) after bisulfite conversion. Data processing followed Bioconductor best-practice workflows, including quality control, normalization, and filtering, with randomization of samples to minimize batch effects [23].

Methylation-based estimates of IL-6 and CXCL9 were obtained using the MethylDetectR platform [24]. Since no validated methylation predictor was available for IL-10, mean methylation across seven CpG sites in the IL-10 promoter region (cg10978799, cg02901679, cg02544380, cg18442793, cg17744604, cg24274865, cg14284394) was calculated. Epigenetic analyses were approved by the Ethics Committee of the Italian National Research Center on Aging (CE INRCA 20031, 04/02/2021).

### Statistical analysis

Continuous variables were expressed as medians with interquartile ranges (IQRs) and categorical variables as counts and percentages. Normality was assessed using the Shapiro–Wilk test. Between-group comparisons (survivors vs. non-survivors) were performed using Wilcoxon rank-sum tests for continuous variables and chi-square tests for categorical variables. Serum IL-6, IL-10, and CXCL9 levels were analyzed across major comorbidities, Frailty Index (FI) categories, and Charlson Comorbidity Index (CCI) strata using Dunn’s test for multiple comparisons. Optimal cut-off values for survival prediction were identified using the Evaluate Cutpoints R package [25]. To facilitate interpretation, cytokines were categorized into low, intermediate, and high levels using optimized time-dependent cut-off values derived from univariable survival analyses. These categories were then combined to generate the composite I3 score (range 3–9).

Associations of cytokines, the I3 score, and FI with 10-year mortality were evaluated using Kaplan–Meier survival curves and log-rank tests, as well as Cox proportional hazards models. Multivariate models were sequentially adjusted to assess independent effects: Model 1 included age, sex, polypharmacy, and CCI (as a continuous variable); Model 2 additionally included laboratory parameters (albumin, eGFR, NLR, AST, ALT, hemoglobin, platelet count, white blood cell count, and glucose); Models 3a–3d further included the categorized FI, analyzed together with IL-6 (Model 3a), IL-10 (Model 3b), CXCL9 (Model 3c), or the I3 score (Model 3 d). Hazard ratios (HRs) and 95% confidence intervals (CIs) were calculated and visualized using forest plots. To account for potential sex differences in biomarker levels and outcomes, analyses were stratified by sex when appropriate.

Multicollinearity among covariates included in the multivariable Cox models was assessed by computing Variance Inflation Factors (VIFs) using auxiliary linear regression models incorporating age, sex, Charlson Comorbidity Index, polypharmacy, and the Frailty Index. All VIF values were below 2, indicating absence of problematic collinearity. Model discrimination for mortality was assessed using time-dependent AUC analyses based on Cox models, with AUCs and 95% CIs estimated using the timeROC R package and pairwise comparisons performed with a DeLong-type test for correlated AUCs. All analyses were conducted using Stata/MP version 18.0 (StataCorp, College Station, TX, USA) and R version 4.3.2. Two-sided p values < 0.05 were considered statistically significant.

## Results

### Clinical characteristics of study subjects

Table 1 summarizes the baseline characteristics of the 1,009 hospitalized older adults included in the study (median age, 84 years; IQR 80–88; 53.2% women). Most admissions were due to neurological or cognitive disorders, cerebrovascular disease, or lung infections (Supplemental Table 2). Patients showed high multimorbidity, frequent polypharmacy, and widespread functional impairment, with more than half meeting criteria for frailty (Frailty Index ≥ 0.20).

**Table 1:** Study subject characteristics

	Total
	***N*** = 1,009
Age (years), median(IQR)	84(80–88)
Gender, *n*(%)	
Male	472(46.8%)
Female	537(53.2%)
1 + lost ADL, *n*(%)	339(33.6%)
1 + lost IADL, *n*(%)	607(60.2%)
CCI, median(IQR)	2(1–3)
Polypharmacy, *n* (%)	641(63.5%)
Frailty Index, *n* (%)	
< 0.10	187(18.5%)
0.10–0.19	359(35.6%)
≥ 0.20	463(45.9%)
Lenght of stay (days), median(IQR)	8(6–11)
Comorbidities	
History of AMI, *n* (%)	143(14.2%)
CHF, *n* (%)	81(8%)
CeVD, *n* (%)	306(30.3%)
Dementia, *n *(%)	246(24.4%)
Depression, *n *(%)	23(2.3%)
COPD, *n* (%)	174(17.2%)
Parkinson, *n* (%)	82(8.1%)
Hypertension, *n *(%)	622(61.6%)
CKD, *n* (%)	216(21.4%)
Anemia, *n *(%)	210(20.8%)
Diabetes, *n *(%)	223(22.1%)
Cancer, *n *(%)	129(12.8%)
Blood Parameters	
Albumin, median(IQR)	3.6(3.2–4.0)
eGFR (BIS1, mL/min), median(IQR)	50.5(36.9–61.5)
NLR, median(IQR)	4.0(2.4–7.4)
AST (U/L), median(IQR)	17(14–24)
ALT (U/L), median(IQR)	13(10–21)
Haemoglobin (g/dL), median(IQR)	12.0(10.5–13.4)
Platelets (n/mm^3^), median(IQR)	204(165–264)
WBC (10^3^ µL), median(IQR)	7.4(5.8–9.7)
Glucose (mg/dL), median(IQR)	102(89–125)

*ADL* Activities of Daily Living, *ALT* Alanine Aminotransferase, *AMI* Acute Myocardial Infarction, *AST* Aspartate Aminotransferase, *BIS1* Berlin Initiative Study 1 equation, *CCI* Charlson Comorbidity Index, *CeVD* Cerebrovascular Disease, *CHF* Congestive Heart Failure, *CKD* Chronic Kidney Disease, *COPD* Chronic Obstructive Pulmonary Disease, *eGFR* Estimated Glomerular Filtration Rate, *IADL* Instrumental Activities of Daily Living, *IQR* Interquartile Range, *NLR* Neutrophil-to-Lymphocyte Ratio, *WBC* White Blood Cell count

Baseline characteristics by sex are reported in Supplemental Table 3; women were older and frailer, whereas men had a higher prevalence of cardiovascular disease and cancer.

Supplemental Table 4 shows the distribution of IL-6, IL-10, CXCL9, and the composite I3 score across frailty and comorbidity categories. All markers and the I3 score were significantly elevated in frail individuals (all *p* < 0.001). IL-10 (*p* = 0.016), CXCL9 (*p* < 0.001), and the I3 score (*p* = 0.004) were also higher in participants with a greater comorbidity burden (CCI ≥ 2), whereas IL-6 did not differ significantly (*p* = 0.189).

### Inflammatory biomarkers, frailty, and long-term mortality

During a total follow-up of 1,897,854 person-days (approximately 5,196 person-years), 833 out of 1,009 participants (82.6%) died. Median follow-up was 50 months (IQR 14–95). Using the inverse Kaplan–Meier method to estimate potential follow-up time, the median follow-up was 127 months (IQR 114–136). The minimum and maximum observed follow-up durations were 3 and 4,558 days, respectively.

Table 2 presents the distribution of IL-6, IL-10, and CXCL9 according to long-term survival status. All three biomarkers were significantly higher in non-survivors (*p* < 0.001). Median IL-6 levels were more than twice as high in deceased patients compared with survivors (16.7 vs. 7.2 pg/mL), with similar trends for IL-10 (4.2 vs. 2.9 pg/mL) and CXCL9 (1864 vs. 1127 pg/mL).

**Table 2:** Distribution of IL-6, IL-10, CXCL9, and I3 score according to long-term survival status

Variable	Total	Survivors	Non-survivors	*p*-value
	(*N* = 1,009)	(*N* = 176)	(*N* = 833)	
IL-6 (pg/ml), median(IQR)	14.1(5.9–39.9)	7.2(3.6–18.9)	16.7(7.1–43.4)	< 0.001
IL-6 (pg/ml), *n*(%)				< 0.001
≤ 3.44	118(11.7%)	39(22.2%)	79(9.5%)	
3.45–11.9	333(33%)	79(44.9%)	254(30.5%)	
≥ 12.0	558(55.3%)	58(33%)	500(60%)	
IL-10 (pg/ml), median(IQR)	4.0(2.7–6.3)	2.9(2.1–4.7)	4.2(2.9–6.6)	< 0.001
IL-10 (pg/ml), *n*(%)				< 0.001
≤ 3.27	388(38.5%)	107(60.8%)	281(33.7%)	
3.28–40.4	606(60.1%)	69(39.2%)	537(64.5%)	
≥ 40.5	15(1.5%)	0(0%)	15(1.8%)	
CXCL9 (pg/ml), median(IQR)	1697(1043–3087)	1127(756.5–1662)	1864(1142–3356)	< 0.001
CXCL9 (pg/ml), *n*(%)				< 0.001
≤ 1706	510(50.5%)	137(77.8%)	373(44.8%)	
1707–5759	400(39.6%)	34(19.3%)	366(43.9%)	
≥ 5760	99(9.8%)	5(2.8%)	94(11.3%)	
I3 Score, median(IQR)	6(5–7)	4(4–6)	6(5–7)	< 0.001
I3 Score categories, *n*(%)				
3–4 points	244(24.2%)	91(51.7%)	153(18.4%)	< 0.001
5–7 points	693(68.7%)	82(46.6%)	611(73.3%)	
8–9 points	72(7.1%)	3(1.7%)	69(8.3%)	

*CXCL9* C-X-C Motif Chemokine Ligand 9, *I3 Score* Inflammaging score based on IL-6, IL-10, and CXCL9, *IL-6* Interleukin-6, *IL-10* Interleukin-10, *IQR* Interquartile Range

To facilitate interpretation, cytokines were categorized into low, intermediate, and high. These categories were then combined to generate the composite I3 score (range 3–9).

Multivariable Cox regression models (Supplemental Table 5) showed that IL-6, IL-10, CXCL9 – categorized using optimized time-dependent cut-off values derived from univariable survival analyses – and the I3 score were independently associated with long-term mortality after adjustment for demographics, comorbidities, polypharmacy, and laboratory parameters. IL-6 ≥ 12.0 pg/mL remained significantly associated with mortality (HR = 1.34, 95% CI 1.03–1.74), and both IL-10 and CXCL9 displayed graded risk increases across categories. The I3 score showed a stepwise association with mortality, with HRs of 1.50 (95% CI 1.22–1.83) for scores 5–7 and 2.58 (95% CI 1.86–3.58) for scores 8–9 versus scores 3–4.

**Fig. 1:**
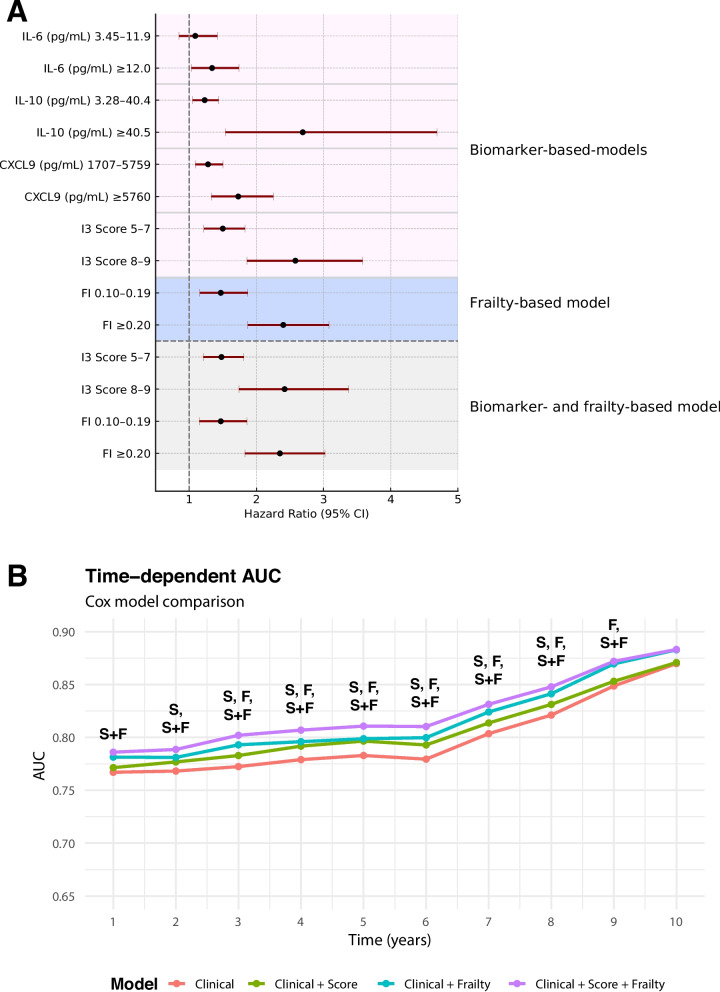
Prognostic value of inflammatory biomarkers and frailty for long-term mortality (**A**) Forest plot of hazard ratios for long-term mortality from multivariable Cox regression models. The top section (pink) represents biomarker-based models (Model 2, adjusted for age, sex, Charlson Comorbidity Index, polypharmacy, albumin, estimated glomerular filtration rate (eGFR), neutrophil-to-lymphocyte ratio (NLR), aspartate aminotransferase (AST), alanine aminotransferase (ALT), hemoglobin, platelet count, white blood cell count (WBC), and glucose), the middle section (blue) represents the frailty-based model (Model 2). The bottom section (gray) represents the integrated model combining biomarkers and frailty (Model 3 d), which corresponds to Model 2 with the addition of both the biomarker-based score and the frailty index. Horizontal lines indicate 95% confidence intervals. **B** Time-dependent ROC curves showing the discriminative performance of models including clinical covariates (“Clinical”), frailty categories (“Frailty”, F: non-frail < 0.10, pre-frail 0.10–0.19, frail ≥ 0.20), and the composite I3 score (“Score”, S, range 3–9). AUCs are plotted annually from year 1 to year 10; pairwise comparisons are reported in Supplementary Table 6. Symbols “S”, “F”, and “S + F” indicate models showing significantly higher AUCs (*p* < 0.05, DeLong test) compared with the clinical model alone

Frailty also emerged as a strong, independent predictor of mortality (Model 2; HR = 1.47, 95% CI 1.16–1.87 for FI 0.10–0.19; HR = 2.40, 95% CI 1.87–3.08 for FI ≥ 0.20 vs. FI < 0.10). When both I3 score and FI were included in the same model (Model 3 d, Fig. 1A, Supplemental Table 5), intermediate and high I3 scores remained independently associated with mortality (5–7, HR = 1.48, 95% CI 1.21–1.81; 8–9, HR = 2.42, 95% CI 1.74–3.37), alongside the FI (FI 0.10–0.19, HR = 1.47, 95% CI 1.15–1.86; FI ≥ 0.20, HR = 2.35, 95% CI 1.83–3.02). In a sensitivity analysis using an alternative FI threshold of ≥ 0.25 [26], the association between frailty and long-term mortality remained strong and consistent. In the fully adjusted model (Model 3 d), FI 0.10–0.24 was associated with increased mortality risk (HR 1.60, 95% CI 1.28–2.00), while FI ≥ 0.25 showed a stronger association (HR 2.60, 95% CI 2.04–3.33).

In time-dependent ROC analyses (Fig. 1B), both the frailty index and the I3 inflammaging score improved discrimination when added individually to the clinical model, which included age, sex, Charlson Comorbidity Index, polypharmacy, and routine laboratory parameters. The AUC improvements associated with FI and I3 were comparable up to approximately the seventh year of follow-up. The combination of both markers further enhanced model performance: the Clinical + FI + I3 model achieved the highest AUCs between years 1 and 7, after which all curves converged. Pairwise comparisons confirmed adding the I3 score to the Clinical + FI model significantly increased AUCs from year 2 through year 7 (Supplemental Table 6).

**Fig. 2:**
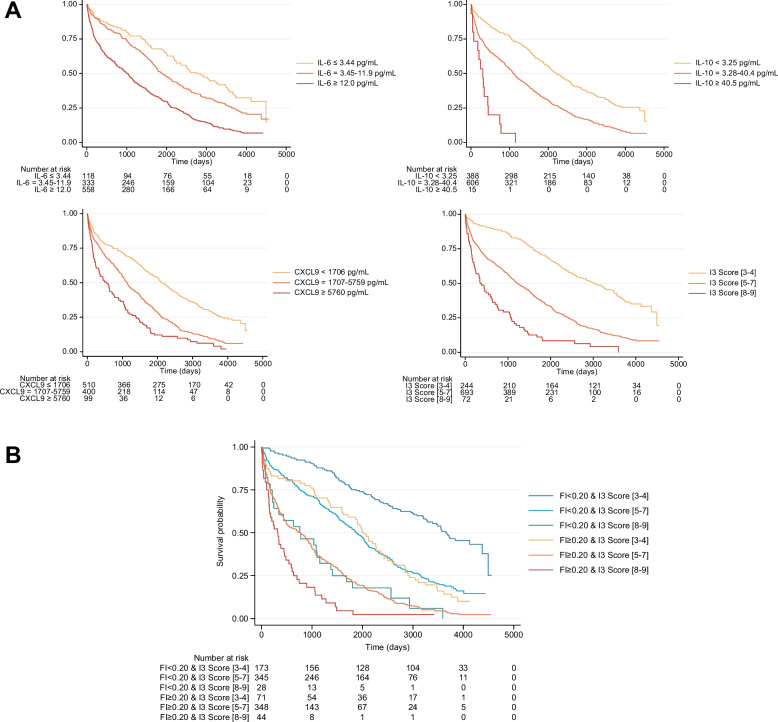
Kaplan–Meier survival analysis of long-term mortality by inflammatory biomarkers and frailty status. **A** Survival curves stratified by IL-6, IL-10, and CXCL9 levels, and by I3 score categories (low: 3–4, intermediate: 5–7, high: 8–9). Groupings were defined using optimized biomarker-specific cut-off values. **B** Combined stratification by Frailty Index (FI < 0.20 vs. ≥ 0.20) and I3 score categories (low, intermediate, high), showing four distinct risk groups for long-term mortality

Kaplan–Meier analysis (Fig. 2A–B) visually confirmed these results. Figure 2A shows progressively shorter survival across increasing cytokine and I3 categories. Figure 2B combines the I3 score with a binary frailty classification (FI < 0.20 vs. ≥ 0.20), illustrating four distinct risk groups: the lowest mortality in patients with both low FI and low I3, and the highest in those with both elevated. Among non-frail individuals, the I3 score still identified higher-risk subgroups, whereas frailty further discriminated risk in those with intermediate I3 levels.

Together, these findings indicate that both inflammaging and frailty independently predict long-term mortality, with comparable effect sizes and complementary prognostic value.

### Epigenetic correlates of IL-6, IL-10, and CXCL9

**Fig. 3:**
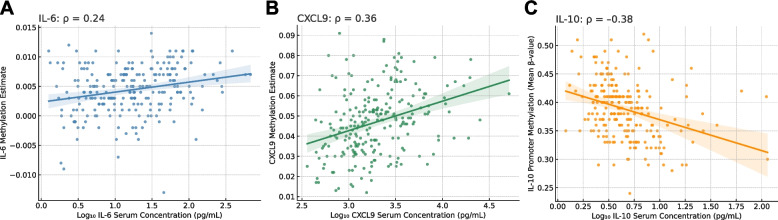
Correlation between cytokine methylation estimates and circulating protein levels. Scatter plots showing the association between methylation-based estimates and measured serum levels of IL-6 (**A**), CXCL9 (**B**), and IL-10 (**C**). IL-6 and CXCL9 methylation scores were derived using the Horvath’s epigenetic predictor, while IL-10 methylation was based on 7 CpG sites within the promoter region. Regression lines with 95% confidence intervals are reported

To explore the role of epigenetic regulation in cytokine expression, we examined DNA methylation profiles in a subset of 237 patients. Serum levels of IL-6 and CXCL9 showed significant positive correlations with their methylation-derived estimates (Fig. 3A–B). Specifically, IL-6 concentrations measured by immunoassay correlated with methylation-inferred IL-6 levels (ρ = 0.24, *p* < 0.001), and similarly for CXCL9 (ρ = 0.36, *p* < 0.001). For IL-10, which lacked a validated methylation-based estimator, we analyzed the mean methylation level across seven CpG sites within the IL-10 promoter region (cg10978799, cg02901679, cg02544380, cg18442793, cg17744604, cg24274865, cg14284394). An inverse correlation was observed between promoter methylation and IL-10 serum concentrations (ρ = –0.38, *p* < 0.001; Fig. 3C).

Collectively, these analyses demonstrated significant associations between circulating cytokine concentrations and DNA methylation profiles of IL-6, IL-10, and CXCL9.

## Discussion

Building on the established link between inflammaging and age-related mortality, this study explored the prognostic value of circulating IL-6, IL-10, and CXCL9 in hospitalized older adults, introducing the composite I3 score as an integrated measure of systemic inflammation. By excluding in-hospital deaths, we focused specifically on long-term mortality, minimizing confounding from acute events. Our findings demonstrate that inflammaging biomarkers, particularly when combined into the I3 score, provide clinically meaningful risk stratification and complement the prognostic information conveyed by frailty.

Among the individual biomarkers assessed, CXCL9 emerged as the most consistent and robust predictor of long-term mortality. Elevated levels were significantly associated with reduced survival, even after full adjustment for clinical and laboratory confounders. CXCL9 is increasingly recognized as a key contributor to age-related immune activation, cardiovascular disease, and cellular senescence. Its involvement in vascular aging and endothelial dysfunction is supported by preclinical models showing that CXCL9 silencing can mitigate senescent phenotypes and restore vascular function [27]. Moreover, recent studies suggest a role for neuroimmune interactions involving vagal tone and macrophage-derived CXCL9 in systemic age-related inflammation [28]. Our findings are in line with previous research linking CXCL9 to frailty, sarcopenia, cognitive impairment, and mortality in both community-dwelling individuals and patients with established diseases [29–33].

IL-6 also demonstrated a significant association with long-term mortality in our cohort, reinforcing its role as a clinically relevant inflammatory marker in aging. Our results extend previous findings by confirming that only elevated IL-6 concentrations (≥ 12.0 pg/mL) were consistently associated with increased mortality risk, even after multivariate adjustment. IL-6 levels also increased with frailty and comorbidity burden, consistent with earlier reports [34–36].

Interestingly, IL-10, traditionally regarded as an anti-inflammatory cytokine, was independently associated with increased mortality, particularly within the intermediate concentration range. Although only a small subset of participants exhibited extreme IL-10 elevations, this group and the more prevalent intermediate group both showed excess mortality risk. Elevated IL-10 may reflect a compensatory response to systemic inflammation, but in the context of immunosenescence and acute stressors it could also contribute to impaired immunity, consistent with the concept of immune paralysis. Elevated IL-10 has been linked to T-cell exhaustion and impaired immune surveillance in sepsis and advanced atherosclerosis [37, 38]. In older adults, even moderate elevations may signal maladaptive immunoregulation and diminished resilience [39], highlighting its potential value as a biomarker of systemic immune dysfunction in aging.

To improve risk prediction beyond individual cytokines, we developed the I3 score, a composite measure integrating IL-6, IL-10, and CXCL9. The I3 score showed a strong, dose–response association with 10-year mortality and retained predictive power even after extensive adjustment for frailty, comorbidity, polypharmacy, and routine laboratory markers. In addition, time-dependent ROC analyses confirmed the additive value of the I3 score for early follow-up risk stratification. Adding the score to clinical covariates and frailty significantly improved mortality prediction from year 2 through year 7 (vs. the clinical + frailty model). The subsequent loss of significance is likely multifactorial (including survivor selection in this very old cohort, signal decay from a single baseline biomarker measurement, rising competing risks, and time-varying effects) and linked to a progressive reduction in individual heterogeneity, leading to convergence of model performance at longer horizons. Overall, these findings support the utility of multi-marker indices for stratifying risk and tailoring care strategies in older patients.

Frailty, assessed via a deficit accumulation–based Frailty Index (FI), was also a strong predictor of mortality. When stratified into three levels, i.e. absence of frailty, pre-frailty and frailty, both pre-frailty and frailty alone identified patients at elevated risk. However, combining FI with the I3 score enhanced risk stratification. This integrated approach identified a continuum of mortality risk, particularly within intermediate groups. Among non-frail individuals, elevated I3 scores distinguished patients with higher mortality risk, while among those with intermediate I3 scores, frailty status further refined prognosis. These findings underscore the complementary nature of frailty and inflammaging, one reflecting clinical and functional decline, the other immune-metabolic dysregulation.

This distinction is particularly relevant in real-world geriatric settings, where patients often present with both chronic comorbidities and acute illness. While the highest-risk and lowest-risk phenotypes were concordant for both frailty and inflammation, the intermediate states benefited most from a combined assessment. This supports a model of aging wherein frailty and inflammaging contribute overlapping but distinct signals, meriting dual measurement for personalized risk prediction.

Although sex differences are well documented in aging and inflammation, no significant sex-based differences in biomarker levels were observed. The acute illness context of hospitalization may obscure baseline immune dimorphism typically seen in community-dwelling populations [40].

To explore upstream regulation of cytokine levels, we analyzed epigenetic profiles in a subset of patients. Circulating IL-6 and CXCL9 levels were correlated with their methylation-based estimates, suggesting transcriptional regulation via DNA methylation. For IL-10, an inverse relationship was observed between promoter methylation at a key CpG site and serum levels. Due to the lack of an established methylation-derived estimator, the use of promoter-specific CpG sites as a proxy may have influenced the IL-10 results, which should therefore be interpreted with caution. Since correlations were modest (< 0.40), these findings provide preliminary data on the association between DNA methylation and cytokine expression. However, stronger evidence is needed to support the hypothesis that circulating cytokine concentrations consistently reflect stable molecular signatures of immune activation.

Strengths of this study include its large, well-characterized cohort, long follow-up, and integration of biomarker, clinical, and epigenetic data. Limitations include its observational and single-center design, potential residual confounding, and the restriction to hospitalized individuals with available samples and preserved consent capacity, possibly underrepresenting severely impaired patients. Moreover, both frailty and inflammatory biomarkers were assessed at a single baseline time point, which limits the ability to capture their dynamic trajectories and to fully distinguish chronic from acute inflammation or transient functional fluctuations. This constraint, together with the specific clinical context of hospitalized older adults, may reduce generalizability to community-dwelling or younger populations and should be considered when interpreting the findings. Finally, cause-of-death information was not available for the large majority of deaths occurring after discharge, preventing cause-specific analyses and limiting deeper interpretation of mortality pathways.

In conclusion, this study supports the integration of IL-6, IL-10, and CXCL9 into a composite I3 score to enhance risk stratification in hospitalized older adults. When combined with frailty assessment, the I3 score is independently associated with long-term mortality prediction and provides a dual lens on the biological and clinical dimensions of aging. These findings contribute to the expanding framework of immunosenescence research and highlight the potential of multi-marker, epigenetically informed tools for guiding individualized geriatric care.

## Supplementary Information

Supplementary Material 1.

## Data Availability

The data that support the findings of this study are available from the corresponding author upon reasonable request.

## Abbreviations

ADL: Activities of Daily Living
ALT: Alanine Aminotransferase
AMI: Acute Myocardial Infarction
AST: Aspartate Aminotransferase
CCI: Charlson Comorbidity Index
CeVD: Cerebrovascular Disease
CKD: Chronic Kidney Disease
CGA: Comprehensive Geriatric Assessment
COPD: Chronic Obstructive Pulmonary Disease
CXCL9: C-X-C Motif Chemokine Ligand 9 (Monokine Induced by Gamma Interferon)
eGFR: Estimated Glomerular Filtration Rate
FI: Frailty Index
HR: Hazard Ratio
ICD-9: International Classification of Diseases, Ninth Revision
IL-6: Interleukin-6
IL-10: Interleukin-10
IQR: Interquartile Range
MDS-AC: Minimum Data Set for Acute Care
NLR: Neutrophil-to-Lymphocyte Ratio
SASP: Senescence-Associated Secretory Phenotype
SD: Standard Deviation
WBC: White Blood Cell
