# Supplementary material for: Association of an inflammaging score based on IL-6, IL-10 and CXCL9, and frailty with long-term mortality in hospitalized older adults
Source: Immun Ageing. 2025 Dec 19;22:56. doi: 10.1186/s12979-025-00553-5 (PMC12750693; doi:10.1186/s12979-025-00553-5)

Association of an inflammaging score based on IL-6, IL-10 and CXCL9, and frailty with long-term mortality in hospitalized older adults

ONLINE SUPPLEMENTAL MATERIALS

Supplemental Table 1. Items evaluated for constructing the Frailty Index. 2
Supplemental Table 2. Distribution of main hospital admission diagnoses in the study population. 3
Supplemental Table 3. Gender differences 4
Supplemental Table 4. Distribution of IL-6, IL-10, CXCL9, and I3 score by Frailty Index and Charlson Comorbidity Index categories 6
Supplemental Table 5. Cox proportional hazards models for long-term mortality 7
Supplemental Figure 1. Kaplan–Meier survival curves according to Frailty Index and combined Frailty Index–I3 score categories. 8

Supplemental Table 1. Items evaluated for constructing the Frailty Index. Each item was scored as 0 (absent) or 1 (present).

**Deficit**
Living in a residential facility for older adults
Behavioral disturbances
Dementia
Hearing impairment
Visual impairment
Dependence to perform hygiene tasks
Dependence on mobility
Dependence in bathing
Dependence on toilet use
Bedridden
Dependence on eating
Urinary incontinence
Fecal incontinence
Heart failure
Acute myocardial infarction
Hypertension
Diabetes
Stroke
Parkinson's disease
Chronic kidney disease
Chronic obstructive pulmonary disease
Osteoarthritis
Cirrhosis
Depression
Cancer
Falls
Weight loss
Dysphagia
Pressure ulcers
Use of medications

Supplemental Table 2. Distribution of main hospital admission diagnoses in the study population.

	n (%)
Diabetes	8 (0.8%)
Metabolism and nutrition disorders	26 (2.6%)
Delirium and other psychiatric disorders	9 (0.9%)
Cerebrovascular disease	137 (13.6%)
Cancer	52 (5.2%)
Anemia	48 (4.8%)
Dementia or other disorders of the nervous system	241 (23.9%)
Heart failure and heart disease	31 (3.1%)
Hypertension or cardiac arrhythmias	48 (4.8%)
Lung infections	124 (12.3%)
Gastrointestinal pathologies	113 (11.2%)
Genitourinary pathologies	61 (6.1%)
Sepsis	23 (2.3%)
Other	88 (8.7%)

Supplemental Table 3. Gender differences

	Total N=1,009	Male N=472	Female N=537	p
Age (years), median (IQR)	84 (80-88)	84 (79-88)	85 (80-89)	0.001
IL-6 (pg/ml), median (IQR)	14.1 (5.94-39.9)	14.95 (6.605-39.35)	13.6 (5.58-40.5)	0.502
IL-6 (pg/ml), n (%)				0.479
≤3.44	118 (11.7%)	50 (10.6%)	68 (12.7%)	
3.45-11.9	333 (33%)	153 (32.4%)	180 (33.5%)	
≥12.0	558 (55.3%)	269 (57%)	289 (53.8%)	
IL-10 (pg/ml), median (IQR)	3.99 (2.71-6.26)	3.925 (2.72-6.09)	4.05 (2.69-6.4)	0.901
IL-10 (pg/ml), n (%)				0.871
≤3.27	388 (38.5%)	182 (38.6%)	206 (38.4%)	
3.28-40.4	606 (60.1%)	282 (59.7%)	324 (60.3%)	
≥40.5	15 (1.5%)	8 (1.7%)	7 (1.3%)	
CXCL9 (pg/ml), median (IQR)	1697 (1043-3087)	1708.5 (1091.5-2981.5)	1659 (1032-3188)	0.972
CXCL9 (pg/ml), n (%)				0.781
≤1706	510 (50.5%)	234 (49.6%)	276 (51.4%)	
1707-5759	400 (39.6%)	189 (40%)	211 (39.3%)	
≥5760	99 (9.8%)	49 (10.4%)	50 (9.3%)	
SCORE, median (IQR)	6 (5-7)	6 (5-7)	6 (4-7)	0.393
SCORE categories, n (%)				0.568
1	244 (24.2%)	107 (22.7%)	137 (25.5%)	
2	693 (68.7%)	330 (69.9%)	363 (67.6%)	
3	72 (7.1%)	35 (7.4%)	37 (6.9%)	
1+ lost ADL, n (%)	339 (33.6%)	131 (27.8%)	208 (38.7%)	0.001
1+ lost IADL, n (%)	607 (60.2%)	265 (56.1%)	342 (63.7%)	0.040
CCI, median (IQR)	2 (1-3)	2 (1-3)	1 (1-2)	0.003
Polypharmacy, n (%)	641 (63.5%)	303 (64.2%)	338 (62.9%)	0.282
Frailty Index, n (%)				0.001
<0.10	187 (18.5%)	106 (22.5%)	81 (15.1%)	
0.10-0.19	359 (35.6%)	175 (37.1%)	184 (34.3%)	
≥0.20	463 (45.9%)	191 (40.5%)	272 (50.7%)	
Lenght of stay (days), median (IQR)	8 (6-11)	7 (5-11)	8 (6-12)	<0.001
1-year mortality, n (%)	241 (23.9%)	118 (25%)	123 (22.9%)	0.436
Long-term mortality, n (%)	833 (82.6%)	399 (84.5%)	434 (80.8%)	0.121

**Comorbidities**				
AMI, n (%)	143 (14.2%)	83 (17.6%)	60 (11.2%)	0.004
CHF, n (%)	81 (8%)	32 (6.8%)	49 (9.1%)	0.171
CeVD, n (%)	306 (30.3%)	139 (29.4%)	167 (31.1%)	0.579
Dementia, n (%)	246 (24.4%)	103 (21.8%)	143 (26.6%)	0.076
Depression, n (%)	23 (2.3%)	8 (1.7%)	15 (2.8%)	0.243
COPD, n (%)	174 (17.2%)	92 (19.5%)	82 (15.3%)	0.077
Parkinson, n (%)	82 (8.1%)	40 (8.5%)	42 (7.8%)	0.705
Hypertension, n (%)	622 (61.6%)	278 (58.9%)	344 (64.1%)	0.093
CKD, n (%)	216 (21.4%)	111 (23.5%)	105 (19.6%)	0.126
Anemia, n (%)	210 (20.8%)	99 (21%)	111 (20.7%)	0.905
Sepsis, n (%)	46 (4.6%)	19 (4%)	27 (5%)	0.446
Diabetes, n (%)	223 (22.1%)	109 (23.1%)	114 (21.2%)	0.476
Cancer, n (%)	129 (12.8%)	78 (16.5%)	51 (9.5%)	0.001
**Blood Parameters**				
Albumin, median (IQR)	3.6 (3.2-4)	3.6 (3.2-4.1)	3.6 (3.2-3.9)	0.097
eGFR (BIS1, mL/min), median (IQR)	50.5 (36.9-61.5)	50.8 (37.5-62.2)	50.5 (36.4-61.4)	0.474
NLR, median (IQR)	4.0 (2.4-7.4)	4.1 (2.5-7.8)	3.8 (2.2-6.9)	0.125
AST (U/L), median (IQR)	17 (14-24)	18 (14.5-25.5)	17 (13-23)	0.019
ALT (U/L), median (IQR)	13 (10-21)	14 (10-22)	12 (10-19)	<0.001
Haemoglobin (g/dL), median (IQR)	12 (10.5-13.4)	12.4 (10.8-14)	11.6 (10.2-12.9)	<0.001
Platelets (n/mm^3), median (IQR)	204 (165-264)	190 (156-237)	223 (173-280)	<0.001
WBC (10^3 µL), median (IQR)	7.43 (5.83-9.74)	7.62 (5.97-10.16)	7.33 (5.7-9.56)	0.087
Glucose (mg/dL), median (IQR)	102 (89-125)	105 (91-130)	101 (88-122)	0.006

Supplemental Table 4. Distribution of IL-6, IL-10, CXCL9, and I3 score by Frailty Index and Charlson Comorbidity Index categories

	**FI < 0.10**	**FI = 0.10-0.19**	**FI ≥ 0.20**	**p**
IL-6 (pg/ml), median (IQR)	10.4 (4.35-27.4)	11.6 (5.08-37.6)	17.9 (7.76-45.1)	**0.000**[b, c]
IL-10 (pg/ml), median (IQR)	3.38 (2.44-5.74)	3.56 (2.41-5.64)	4.38 (2.92-7.04)	**0.000**[b, c]
CXCL9 (pg/ml), median (IQR)	1366 (878-2254)	1484 (986-2670)	2051 (1194-3633)	**0.000**[b, c]
I3score median (IQR)	5 (4-6)	6 (4-7)	6 (5-7)	**0.000**[b, c]
	**CCI = 0**	**CCI = 1**	**CCI ≥ 2**	**p**
IL-6 (pg/ml), median (IQR)	16.5 (4.93-40.7)	12.6 (5.45-30.7)	14.9 (6.74-43.4)	0.189
IL-10 (pg/ml), median (IQR)	4.10 (2.52-6.75)	3.6 (2.44-5.55)	4.14 (2.85-6.43)	**0.016**[f]
CXCL9 (pg/ml), median (IQR)	1338 (867-2313)	1583 (1018-2754)	1869 (1141-3576)	**0.000**[e, f]
I3 score median (IQR)	6 (4-6)	6 (4-7)	6 (5-7)	**0.004**[e, f]

a $p < 0.05$ for Frailty Index < 0.10 vs Frailty Index = 0.10-0.19

b $p < 0.05$ for Frailty Index < 0.10 vs Frailty Index ≥ 0.20

c $p < 0.05$ for Frailty Index = 0.10-0.19 vs Frailty Index ≥ 0.20

d $p < 0.05$ for CCI = 0 vs CCI = 1

e $p < 0.05$ for CCI =0 vs CCI ≥ 2

f $p < 0.05$ for CCI =1 vs CCI ≥ 2

CCI: Charlson Comorbidity Index; FI=Frailty Index

Supplemental Table 5. Cox proportional hazards models for long-term mortality

	Model 1 HR (95%CI)	Model 2 HR (95%CI)	Model 3a HR (95%CI)	Model 3b HR (95%CI)	Model 3c HR (95%CI)	Model 3d HR (95%CI)
IL-6 (pg/mL)						
≤3.44						
3.45-11.9	1.12 (0.87-1.44)	1.09 (0.85-1.42)	1.07 (0.83-1.39)	-	-	-
≥12.0	1.64 (1.28-2.10)	1.34 (1.03-1.74)	1.33 (1.02-1.73)	-	-	-
IL-10 (pg/mL)						
≤3.27						
3.28-40.4	1.49 (1.29-1.73)	1.23 (1.05-1.44)	-	1.21 (1.03-1.42)	-	-
≥40.5	3.75 (2.19-6.41)	2.69 (1.54-4.69)	-	2.59 (1.48-4.51)	-	-
CXCL9 (pg/mL)						
≤1706						
1707-5759	1.33 (1.14-1.54)	1.28 (1.09-1.50)	-	-	1.24 (1.05-1.45)	-
≥5760	1.95 (1.53-2.47)	1.73 (1.33-2.25)	-	-	1.69 (1.30-2.19)	-
SCORE						
3-4						
5-7	1.72 (1.43-2.08)	1.50 (1.22-1.83)	-	-	-	1.48 (1.21-1.81)
8-9	3.33 (2.46-4.51)	2.58 (1.86-3.58)	-	-	-	2.42 (1.74-3.37)
Frailty index						
<0.10						
0.10-0.19	1.36 (1.07-1.72)	1.47 (1.16-1.87)	1.50 (1.19-1.90)	1.47 (1.15-1.86)	1.46 (1.15-1.86)	1.47 (1.15-1.86)
≥0.20	2.30 (1.79-2.94)	2.40 (1.87-3.08)	2.42 (1.89-3.11)	2.37 (1.85-3.05)	2.36 (1.84-3.04)	2.35 (1.83-3.02)

Model 1: Adjusted for age, sex, Charlson Comorbidity Index, and polypharmacy.

Model 2: Model 1 plus adjustment for routine laboratory parameters: albumin, estimated glomerular filtration rate (eGFR), neutrophil-to-lymphocyte ratio (NLR), aspartate aminotransferase (AST), alanine aminotransferase (ALT), hemoglobin, platelet count, white blood cell count (WBC), and glucose.

Models 3a–3d: Model 2 with the addition of frailty index, analyzed together with: 3a: IL-6 categories, 3b: IL-10 categories, 3c: CXCL9 categories, 3d: the I3 score,

Bold values indicate statistically significant hazard ratios ($p < 0.05$).

Supplemental Figure 1. Kaplan–Meier survival curves according to Frailty Index and combined Frailty Index–I3 score categories. A. Survival curves stratified by Frailty Index (FI) categories: non-frail (FI <0.10), pre-frail (FI 0.10–0.19), and frail (FI ≥0.20). B. Survival curves stratified by the combination of Frailty Index categories and I3 score levels (low, intermediate, high).

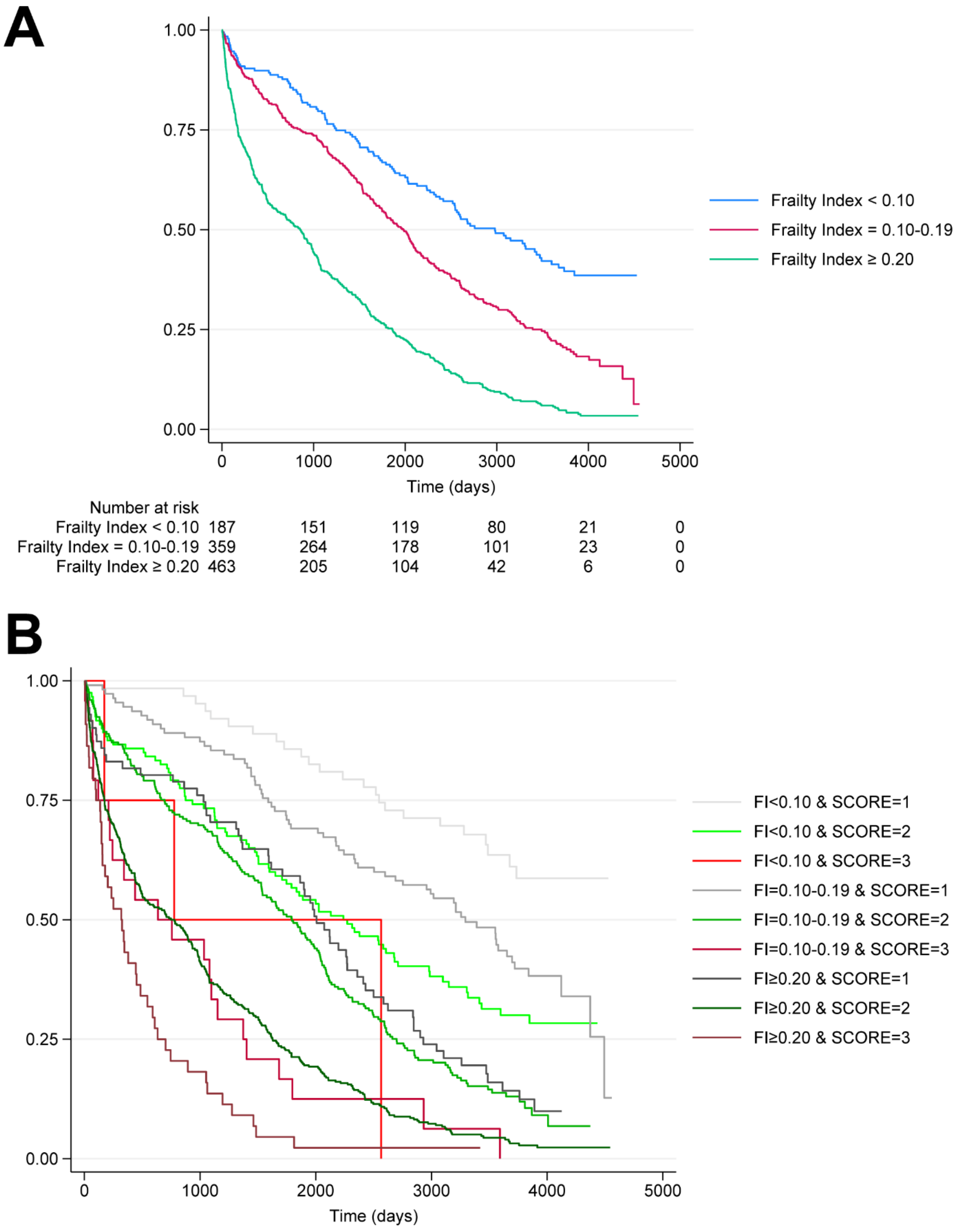

Supplement: Supplementary file 1 — Supplementary Material 1. [file 12979_2025_553_MOESM1_ESM.pdf]
